# What Do Antenatal Women Want From Their Antenatal Education? A National Survey

**DOI:** 10.1007/s10995-025-04048-z

**Published:** 2025-02-03

**Authors:** Eva Larkai, Anna Davies, Miriam Toolan, Mary Lynch, Rachel Plachcinski, Michael Larkin, Abigail Fraser, Christy Burden, Abi Merriel

**Affiliations:** 1 Academic Women’s Health Unit, Bristol Medical School, University of Bristol, Bristol, UK; 2 National Childbirth Trust, Bristol, UK; 3 Department of Psychology, Aston University, Birmingham, UK; 4 Bristol Medical School, Population Health Sciences, University of Bristol, Bristol, UK; 5 Present Address: Centre for Women’s Health Research, Department of Women’s and Children’s Health, Institute of Life Course and Medical Sciences, University of Liverpool, Crown Street, Liverpool, L8 7SS UK; 6 Department of Women’s and Children’s Health, North Bristol NHS Trust, Bristol, UK

**Keywords:** Antenatal education, Birth preparation, National health Ssrvice (NHS), Perinatal mental health

## Abstract

**Introduction:**

Antenatal education (ANE) equips pregnant women with knowledge and skills for pregnancy, birth, and the postnatal period. It should facilitate preparation for the whole spectrum of the maternal journey and empower women to make informed decisions. This study aimed to explore the antenatal education needs and preferences of women who are currently pregnant or planning a pregnancy.

**Methods:**

A UK wide cross-sectional survey was conducted (September 2019 to July 2020), recruiting women living in the UK, above 16, who were currently pregnant or planning a pregnancy. The survey gathered demographic information, details of current or planned class attendance, preferred ANE providers and desired skills and information. Quantitative data were analysed descriptively, and free-text responses underwent thematic analysis.

**Results:**

Of 553 participants included in the analyses, 77% preferred free National Health Service (NHS) classes and 60% planned to attend multiple class types, including paid options. Participants valued practical skills, particularly for labour and the postpartum period, and actively sought perinatal social networks. Multiparous women were less likely to attend classes, citing prior experience or practical barriers. Despite high interest in NHS classes, regional variations in availability and limited accessibility were noted.

**Conclusion:**

NHS antenatal classes are a trusted source of ANE, forming a core element of many women’s antenatal journey. However, inconsistent provision highlights the need for a standardised, comprehensive curriculum. Flexible delivery models and tailored content are crucial to address diverse needs, particularly for multiparous women and underrepresented groups. Enhanced accessibility could reduce inequalities in ANE provision and improve maternal outcomes.

**Supplementary Information:**

The online version contains supplementary material available at 10.1007/s10995-025-04048-z.

## Significance

What is Already Known on this Subject?

What this Study Adds?

Antenatal education (ANE) equips women with knowledge and skills for pregnancy, birth, and the postnatal period, fostering informed decision-making and preparation. However, ANE delivery across the UK is inconsistent, with no standardised curriculum, resulting in regional disparities and barriers to access.

This study identifies women’s preferences for practical skills, trimester-appropriate content, and social support through NHS classes, while highlighting gaps in accessibility and delivery. It advocates for a standardised, flexible ANE model to address diverse needs, reduce inequalities, and enhance maternal outcomes across the UK.

**Supplementary Information:**

The online version contains supplementary material available at 10.1007/s10995-025-04048-z.

## Introduction

Childbirth is a significant event for every family. Women generally anticipate positive experiences that meet or exceed their positive sociocultural beliefs and expectations (Ahlden et al., 2012; Downe et al., 2018; Hall & Wittkowski, 2006). However, women are often also aware of the uncertainties surrounding what lies ahead for their pregnancy, birth and motherhood (Department of Health, 2011). Antenatal education provides an essential opportunity for expectant mothers/parents and their partners to develop strategies to deal with these events and make informed decisions (Brixval et al., 2015). In the context of this study, antenatal education encompasses any formal classes or programmes that provide information and skills to expectant parents, incorporating topics on pregnancy, birth preparation, childbirth, and early parenting.

In the UK ANE is recommended by the National Institute for Health and Care Excellence (NICE) (National Institute for Health and Care Excellence (NICE), 2021) and available within the National Health Service (NHS). It is frequently delivered by NHS midwives, free of charge. However, whether these classes are provided, and the content delivered, is at the discretion of individual Trusts, with no curriculum mandated by the NHS or bodies such as NICE. Due to this, NHS Trusts implement ANE in a variety of ways with some offering several hours of classes and others offering more limited antenatal education opportunities. It has been highlighted that many NHS services encounter difficulties providing this broad-based curriculum, for example due to inadequate provision of further training and support to allow health professionals to effectively deliver this education (Department of Health & Social Care, 2011). Within the NHS, classes often begin around 30–32 weeks of pregnancy and cover a range of topics (National Health Service (NHS), 2021). Women can expect to be informed about health and wellbeing during pregnancy, the birth process, including pain relief and obstetric interventions, and learn skills to care for their newborn (Brixval et al., 2015; NHS, 2021). Antenatal education can also be accessed in the private sector, where antenatal preparation classes may be delivered by clinically or non-clinically trained providers, for example by the National Childbirth Trust (NCT), hypnobirthing providers, and through exercise classes such as antenatal yoga, Pilates and swimming classes.

Antenatal education can play a powerful role in informing and shaping the preconceptions of expectant birthing parents, for those experiencing their first pregnancy (Department of Health & Social Care, 2011; Gagnon & Sandall, 2007; Murphy Tighe, 2010). Some qualitative studies suggest that antenatal education may sometimes encourage the belief that births free from technical, surgical or pharmacological intervention are 'normal' or 'natural', potentially leading to the association of any required interventions with feelings of failure or disappointment (Davies et al., 2023; Downe et al., 2018; Shub et al., 2012). Consequently, when positive expectations are unmet, whether derived from social or cultural narratives or the influence of people within their personal network, women may experience negative feelings, which can result in short- and long-term including fear of childbirth, increased risk of post-partum depression and potential impacts upon the birthing person’s relationship with her new-born baby and partner (Garthus-Niegel et al., 2018; Hall & Wittkowski, 2006; Henriksen et al., 2017; NHS England, 2016; Nilsson et al., 2012). Antenatal education therefore plays a dual role: providing supportive information that aligns with women’s hopes for their birth experience while also setting up a realistic understanding of the spectrum of care that may be required. By offering balanced and accurate information, antenatal education can help dispel misconceptions and empower women to make informed decisions about their care. This balanced approach is essential, as childbirth is inherently unpredictable, and even pregnancies initially deemed low risk may require interventions. For women likely to require these interventions, antenatal education can offer a supportive framework, helping to prepare them for different possibilities in a way that respects their preferences without inducing fear.

Antenatal education should be comprehensive, tailored and accessible to all women (Downe et al., 2019). However, some expectant parents have described their antenatal education to be prescriptive and inflexible, failing to accommodate their needs or the needs of their babies and wider family (Department of Health & Social Care, 2011; Marmot, 2010; Svensson et al., 2008). Barriers to attendance include practical concerns, such as a lack of awareness of their availability, as well as undesirable antenatal education delivery methods such as, didactic teaching methods. Perceptual barriers have also been described, including a lack of perceived importance of the benefit of antenatal education, (Murphy Tighe, 2010; Smith, 2009).

To enable the development and delivery of high-quality antenatal education, we aimed to hear from pregnant women or women planning pregnancy about what their antenatal education needs are and to provide suggestions for the improvement of antenatal education provision in the UK.

## Methods

### Design

Between September 2019 and July 2020, we conducted a UK wide online cross-sectional survey with both multiple choice and free-text questions. The survey contained 20 core items, and gathered demographic data, information about the classes women were currently attending or planning to attend, what information and practical skills they would like to receive, and which antenatal education provider they would prefer to learn from. The survey was piloted for usability and understanding (Supplementary Data 1).

### Participants and Recruitment

Social media-enabled health research recruitment was used to enhance recruitment of a diverse group of survey participants (Arigo et al., 2018). To be eligible for inclusion in the study, women also had to be currently pregnant or planning a pregnancy, had to reside in the UK and be over the age of 16. Informed consent was provided by each participant prior to completing the questionnaire and they were made aware of how their anonymity would be protected.

The survey recruitment graphic and hyperlink were advertised on social media platforms including Facebook, Instagram, and Twitter, and in relevant online pregnancy forums. For groups restricting access to solely mothers, group administrators and moderators were contacted to disseminate the survey. Furthermore, specialists providing antenatal education in locations across the UK, including midwives, hypnobirthing coaches and doulas were identified through the NHS ‘Find Antenatal classes’ search function (NHS, 2021), and contacted to circulate the survey to women within their networks. Participants who accessed the survey were encouraged to refer others to the survey, thus employing a snowball sampling strategy (Parker et al., 2019).

### Analysis

The survey was hosted via the web-based Research Electronic Data Capture (REDCap) software (Harris et al., 2009). Data were transferred into Microsoft Excel for analysis. Responses where participants had only completed their demographic details were excluded, such that participants completing at least one of the core survey items were included in the analysis. We compared those women who reported that they would not be attending antenatal classes with those that were currently attending or planned to attend classes, to determine if demographic differences could be identified between these two groups. To examine the relationship between antenatal class attendance (‘attenders’, ‘non-attenders’) and gravidity (primigravidae, multigravidae), we used a Chi-square test after verifying its assumptions (the sample consisted of independent observations, and the count in each cell was larger than 5). A p-value less than 0.05 will indicate statistical significance.

For quantitative data, counts and percentages of participants responding to each question were calculated. Some participants did not answer every question, and this is reflected in the variation seen in the denominators.

Thematic analysis of the qualitative data was conducted. This involved an iterative process of reading and re-reading the data, and coding it into broad themes (Braun, 2006). We employed an inductive coding procedure, where the themes were identified from the data in relation to the questions asked. EL conducted data analyses, with a second researcher (AD/AM) reviewing codes and themes to ensure validity (Press, 2005). Quotes relating to each question and the themes relating to them were discussed by the research team and summarised for each topic using tables and relevant quotes.

## Results

### Demographic Details

A total of 553 participants completed at least one core survey item. Of these, 138 (25%) indicated that they were not attending or planning to attend antenatal classes, and this is where they finished the survey. These participants are referred to as ‘antenatal class non-attenders’. Overall, there were 415 participants who were currently attending (20% (84/415)) or hoping to attend (80% (331/415)) an antenatal class. These participants are referred to as ‘antenatal class attenders’.

The majority of those in the antenatal class attenders group resided in England and Wales and were in the 30–39 age group (243/415, 58.6%). Most were in their first pregnancy (222/415, 53.5%). A small proportion were not pregnant when completing the survey (71/415, 17.1%) and were categorised as ‘pre-conceptual’. The demographic characteristics of the ‘antenatal class attenders’ and ‘antenatal class non-attenders’ are compared in Table 1. These two groups were largely comparable across all characteristics, however gravidity emerged as a key difference. A chi-square test demonstrated an association between antenatal class attendance and gravidity (X^2^ = 92.880; df = 2; *p* =  < 0.001). The ‘antenatal class attenders’ were more likely to be primigravidae, whereas the ‘antenatal class non-attenders’ were more likely to be multigravidae.

**Table 1:** Sample characteristics

	Antenatal class attenders	Antenatal class non-attenders
	No. (% of total N)	No. (% of total N)
*Total N*	415 (100.0)	138 (100.0)
*Age*		
*18 to 24*	39 (9.4)	8 (5.8)
*25 to 29*	113 (27.2)	35 (25.4)
*30 to 39*	243 (58.6)	91 (65.9)
*40 to 44*	20 (4.8)	4 (2.9)
*Number of children*		
*0 children and not pregnant*	47 (11.3)	0 (0.0)
*0, currently pregnant*	222 (53.5)	25 (18.1)
≥ *1 children*	146 (35.2)	113 (81.9)
*Pregnancy status*		
*Currently pregnant*	344 (82.9)	128 (92.8)
*Not currently pregnant*	71 (17.1)	10 (7.2)
*Region of residence*		
*Wales*	172 (41.4)	75 (54.3)
*England*	241 (58.1)	62 (44.9)
*Ireland*	2 (0.5)	0 (0.0)
*Unanswered*	1 (0.2)	1 (0.7
*Highest educational qualification*		
*Postgraduate degree or equivalent*	167 (40.2)	47 (34.1)
*Undergraduate degree or equivalent*	138 (33.3)	43 (31.2)
*A-levels or equivalent*	64 (15.4)	21 (15.2)
*GCSE’s or equivalent*	26 (6.3)	16 (11.6)
*Vocational qualification*	14 (3.4)	6 (4.3)
*Prefer not to say*	3 (0.7)	3 (2.2)
*Other*	3 (0.7)	2 (1.4)
*Place of birth*		
*UK*	367 (88.4)	125 (90.6)
Elsewhere*	48 (11.6)	13 (9.4)
*Ethnic group*		
*White*	384 (92.5)	125 (90.6)
*Asian or Asian British*	7 (1.7)	3 (2.2)
*Black or Black British*	5 (1.2)	3 (2.2)
*Mixed*	15 (3.6)	3 (2.2)
*Chinese*	1 (0.2)	1 (0.7)
*Prefer not to say*	0 (0.0)	1 (0.7)
*Other*	3 (0.7)	2 (1.4)

### ***What Classes Do Women Plan to Attend? (***Fig. 1***)***

**Fig. 1:**
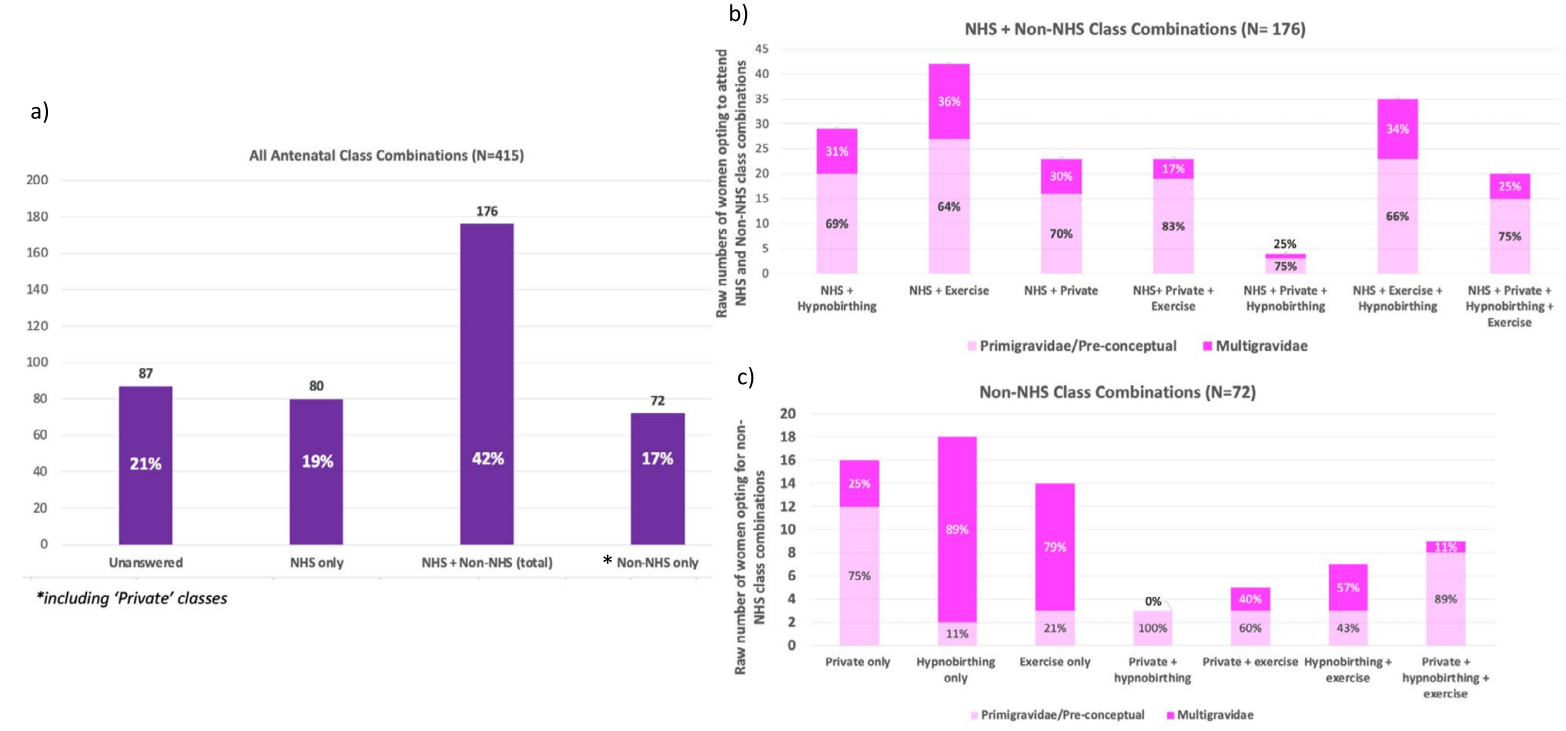
The counts of women who currently attend or plan to attend different combinations of antenatal classes. The ‘Exercise’ category is inclusive of Aqua aerobics, Pregnancy Yoga and Pilates. The ‘Private’ category comprises National Childbirth Trust (NCT) classes, and non-NCT private classes. a All antenatal class combinations (b) NHS + non-NHS class combinations (c) Types of classes women who do not currently attend NHS classes plan to attend. The percentage of those who are primigravidae/pre-conceptual vs multigravidae are presented (1b, 1c)

Women attended or planned to attend different combinations of antenatal classes. Eighty-seven women did not answer this question. Sixty-one percent (200/328) planned to attend more than one type of class. Women who were primigravidae or pre-conceptual were more likely to attend more than one type of class (70% (140/200)) in comparison to women who were multigravidae (30% (60/200)). Of those who planned to attend only one type of antenatal class (39% (128/328)), most planned to attend NHS antenatal classes (63% (80/128)).

Overall, 78% (256/328) of women aimed to or already attended NHS classes. Where additional antenatal classes were sought alongside those provided by the NHS, exercise classes were most frequently chosen. Amongst women who do not currently attend or plan to attend any NHS antenatal classes specifically (*n* = 72), hypnobirthing alone was the most popular class, followed by NCT or non-NCT private classes (Fig. 1c).

### When Do Women Want to Attend Antenatal Classes?

Sixty-four percent of participants wanted to begin NHS classes between 25–35 weeks’ gestation, whilst 29% wanted to start between 13–24 weeks’ gestation. Qualitative responses revealed substantial variation in participants’ desires and expectations for the provision of specific types of antenatal education at different timepoints. Despite this variation, there was some consensus. For example, the majority felt it was most relevant to receive general information, information specific to pregnancy and start exercise classes earlier in pregnancy, whereas they largely preferred to receive advice relevant to the postnatal period and practical skills, such as breastfeeding, later in pregnancy or in the third trimester (Table 2).

**Table 2:** The timepoints in pregnancy that participants most commonly deemed appropriate to receive antenatal education on specific topics. There were 174 free-text responses to the following question: “Which classes would you like to attend at which times?”. These responses were categorised by the timepoint stated by the participant (i.e. first trimester, “mid-pregnancy”). The total number of free-text responses relevant to the type of antenatal education, and the timepoint desired has been noted as ‘No. of relevant responses’. One representative free-text response is included for each timepoint category

	First Trimester (1-12wks)	Second Trimester (13-26wks)	Third trimester (27wks +)	Earlier in pregnancy	Mid- pregnancy	Later in pregnancy
**Labour and birth preparation**	*“Pelvic floor educational classes earlier in pregnancy, birthing options in the 1st 2 trimesters and any other information could be later on in pregnancy” (multigravida)*	*“… Second trimester information about birth choices and labour…” (primigravida)*	*“What to expect in labour and after birth in third trimester (31–36 weeks)…” (primigravida)*	*“…birth options at an earlier stage and then more specific information on breastfeeding and looking after a baby closer to the time of giving birth.” (multigravida)*	*“Pregnancy classes earlier Labour and birth in the middle Baby care skills last” (primigravida)*	*“General info first, labour and birthing techniques later, yoga all throughout” (primigravida)*
*No. of relevant responses*	1	14	13	9	4	12
**Postnatal advice and practical skills e.g. breastfeeding**		*“… Breastfeeding- from week 25/28…” (multigravida)*	*“…Breastfeeding and postnatal info, including basics of caring for a newborn, things to discuss with partner in third trimester sometime.” (primigravida)*	*It would be helpful to have information re delivery options and post natal care earlier on in pregnancy—especially for first time mothers to as to ease anxieties of what is to come! (primigravida)*	*“…Breastfeeding mid-end so fresh in mind” (multiigravida)*	*“Classes re: postnatal care and caring for baby to be held later on in pregnancy so that it's fresh in your mind after the birth.” (primigravida)*
*No. of relevant responses*	0	3	*22*	*3*	*1*	*29*
**Exercise and fitness**	*“Hyponobirthing in third trimester Yoga from first trimester” (primigravida)*	*“From second trimester—pregnancy yoga, relaxation *etc.*…” (multigravida)*		*“… yoga / pilates from early on to get benefits throughout pregnancy” (multigravida)*		*“… at later stage classes with lactation consultants, aqua classes…”*
*No. of relevant responses*	*8*	7	0	16	0	1
**Information specific to pregnancy**	*“Early pregnancy information in the first trimester” (primigravida)*	*“info on maintaining a healthy pregnancy and complications that could arise…” (primigravida)* *“…What's happening and what you can do to keep safe/what's normal… baby position the 2nd trimester. I had no information about baby position until it was too late and baby was breech…” (primigravida)*		*“Learning about pregnancy, issues you could experience and labour earlier on…” (multigravida)* *“I think it would be handy to have early pregnancy classes for first time mums to give them some support and an idea of what to expect” (primigravida)*		
*No. of relevant responses*	*3*	*5*	*0*	*13*	*0*	*0*
**Hypnobirthing**	*“Hypnobirthing and yoga from 12 weeks…” (primigravida)*	*“Hypbobirthing earlier in the pregnancy, perhaps 20 weeks onwards, to practice the technique and get all the benefits it offers in pregnancy.” (multigravida)*	*“…I am about to start my hypnobirthing course at 33 weeks and this feels about right to me, I think anything from about 30 weeks when I was starting to feel more prepared and thinking more about the actual labour!” (primigravida)*	*“Hypnobirthing as early as possible to get in practice…” (primigravida)*	*“Hypnobirthing mid pregnancy to allow time to practice…” (multigravida)*	*“Hypnobirthing neare[r] birth” (multigravida)*
*No. of relevant responses*	*1*	*14*	*6*	*7*	*2*	*2*
**Pelvic floor exercises**	*“Yoga/Pilates and Pelvic floor exercises from 12 week…” (primigravida)*	*“Pelvic floor exercises—from 13–24 weeks…”(primigravida)*	*“… Anything about giving birth or pelvic floor 29–32 weeks” (primigravida)*	*“Pelvic floor educational classes earlier in pregnancy…” (multigravida)*		
*No. of relevant responses*	*1*	*2*	*1*	*2*	*0*	*0*
**General information i.e. physical and mental wellbeing**	*“Support for pregnancy physical and mental health from first trimester onwards…” (primigravida)*			*“It would be useful to have general information about keeping healthy in pregnancy and birth options at an earlier stage…” (multigravida)*		
*No. of relevant responses*	*1*	*0*	*0*	*15*	*0*	*0*
**Relaxation and breathing techniques**		*“… breathing techniques and anxiety reducing methods in second trimester…” (primigravida)*	*“… breathing exercise after 30 weeks” (primigravida)*			*“Depending on stage of pregnancy different symptoms and things are going on. It would be helpful to know what happens at all midwife appointments early on, but in later classes learn breathing techniques and hospital bag packing *etc.*” (primigravida)*
*No. of relevant responses*	0	*4*	1	0	0	1
**Preferences on timing of antenatal classes in general**	*“Antenatal 12–20 weeks with refresher at 35…” (primigravida)*	*“A lot of women give birth prematurely prior to ever starting any antenatal classes a basic class of birth and what to expect should be given between 20–24 weeks.” (multigravida)*	*“…Then the antenatal classes later in pregnancy, perhaps 30 or so weeks onwards so all the practical information is fresher in the mind.” (multigravida)*	*“I feel most classes are provided too late on the nhs, I'd rather start them early to better prepare /practice than when I'm preparing for my baby's arrival.“ (primigravida)*		
*No. of relevant responses*	*1*	8	5	6	0	1

### ***Why Do Women Want to Attend Particular Antenatal Classes? (***Fig. 2***)***

**Fig. 2:**
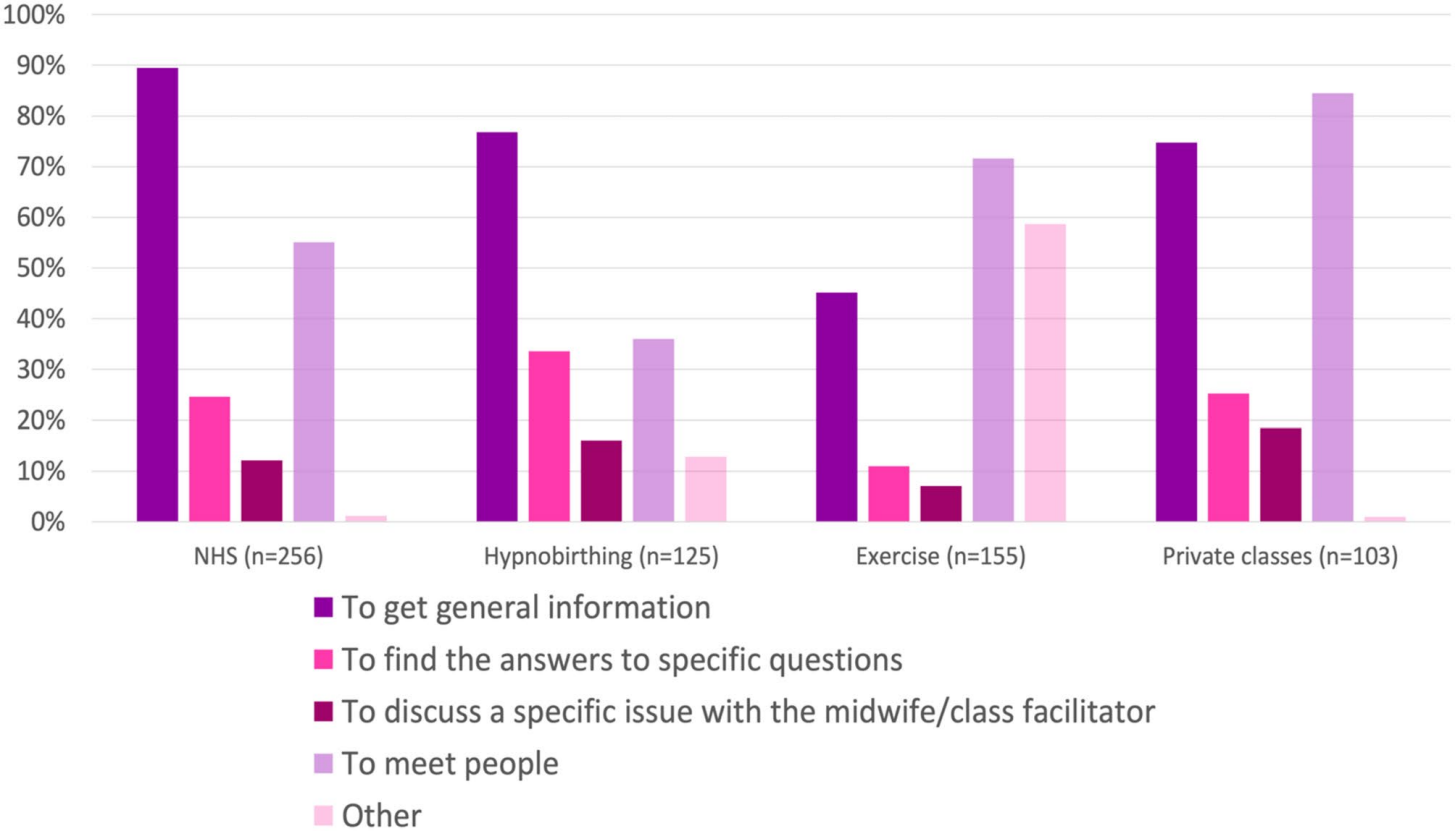
The reasons why women currently attend or plan to attend NHS, hypnobirthing, Exercise (pregnancy yoga/Pilates, aqua aerobics) or private (NCT and non-NCT private) classes

Women reported attending different types of antenatal class with different objectives. Ninety percent of women (n = 229/256) who currently attend or plan to attend NHS classes attended with the intention of obtaining general information, and 55% (141/256) reported that they attended to improve their social networks through meeting others.

This contrasted with the women who planned to attend private classes (84% (87/103)) or exercise classes (Pregnancy yoga/Pilates/Aqua aerobics 72% (111/155)) who predominantly attended to meet others (Fig. 2). For example, one participant stated “…*I signed up to NCT to meet people similar to me who would be having a baby around the same time—for support, social contact *etc*. when I'm on maternity leave.”*

Women choosing to attend hypnobirthing classes (n = 125) had specific aims that related to increasing confidence with hypnobirthing techniques; to improve pain management in pregnancy and labour; to make the process of pregnancy, labour and birth easier; to retain a sense of control; and to improve management of stress and anxiety. Reasons for attending exercise classes were: for general health and wellbeing; for fitness and safe exercise; flexibility; relaxation and enjoyment.

### ***What Do Women Hope to Gain From Antenatal Classes? (***Fig. 3***)***

**Fig. 3:**
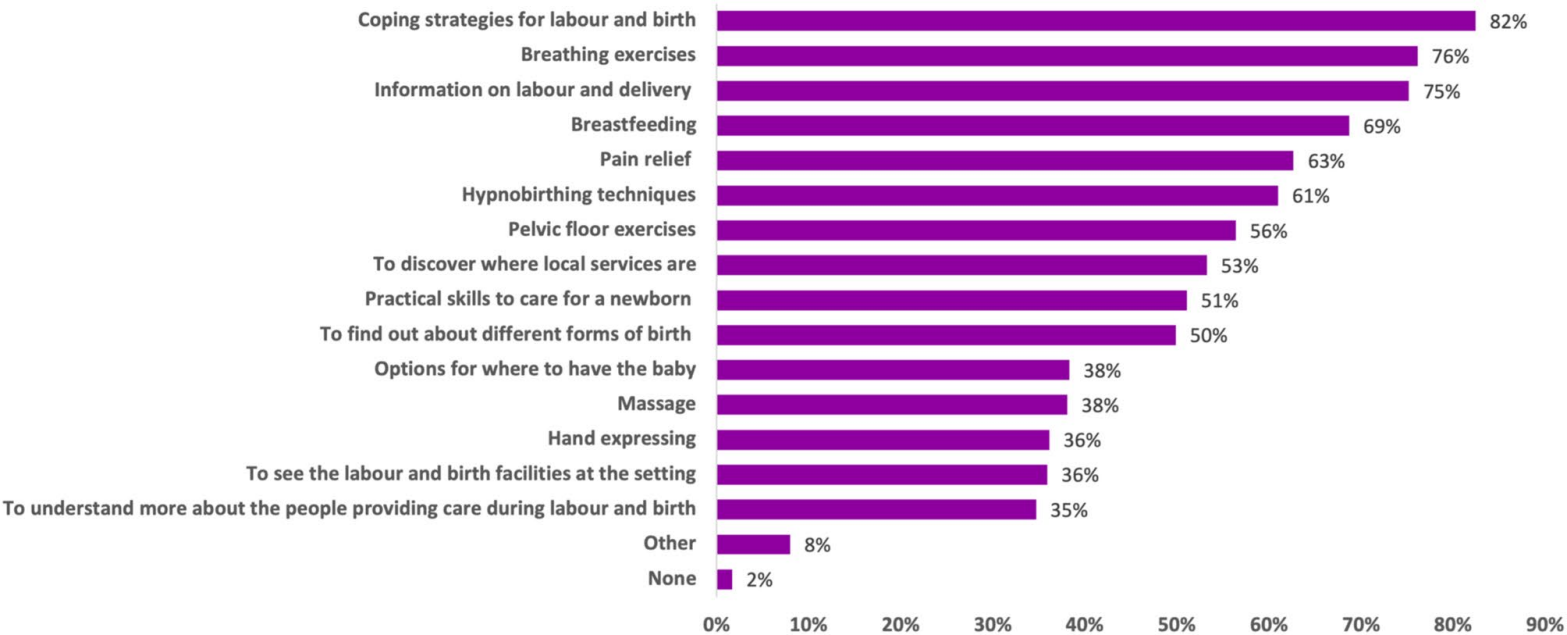
The information and practical skills women are hoping to gain from the class(es) that they attend (*n* = 415

When asked what specific information and skills they hoped to gain from antenatal classes, respondents desired to be equipped with a range of skills and a breadth of information. Information about coping strategies, breathing exercises and labour and delivery, were desired by 82%, 76% and 75% of women respectively. Information and skills relevant to the postnatal period, such as breastfeeding, were also of high importance (69%). In free text responses, several women identified breastfeeding as a challenging practical skill and desired further information on techniques and overcoming difficulties. One respondent felt that this would support women breastfeeding for a longer period. She wrote *“more about breastfeeding difficulties to prepare women better. I think women underestimate how hard it is and don't have a true idea of what responsive feeding is really like. I think with more transparency women would more likely continue breastfeeding for longer”*.

In free-text responses, participants provided several suggestions for additional information and practical skills that would be helpful to receive during antenatal classes. Some respondents wanted information about mental and physical wellbeing during and following pregnancy including information on antenatal nutrition, mental health support, with some mentioning concerns about postnatal depression, managing anxiety and identifying concerns and complications during pregnancy and the birth process (Table 3). Importantly, a recurrent theme that emerged from the data was the desire for the provision of this information to be relevant and appropriate to the individual woman’s stage of pregnancy, or to the issues they were encountering. For example, one respondent wrote *“It would be great to be able to choose. E.g., 'I'm at the stage of my pregnancy now where I am thinking about breastfeeding/where to give birth/managing pregnancy discomfort' *etc*. and being able to attend the session that covered that. For different people these issues might be more or less important at different times during their pregnancy.”* (Table 3).

**Table 3:** Themes emerging from the data and supporting quotes from participants

Do you have any other views you would like to share about what should be included in antenatal classes and how it could be included?	
Theme	Supporting data
Greater accessibility	*“I feel that the access to NHS antenatal varies greatly in areas and we have only been offered 1 session …” (primigravida)* *“Me and my partner have attended midwife-led antenatal classes with [Non-NCT Private class] which have provided excellent balanced and evidence-based information without judgement which have been fantastic. The fact that you have to pay to access this information excludes lots of prospective parents who would really benefit from this information and support which is wrong- this should be available to all…” (primigravida)*
Improved postnatal support	*“I believe there should be more focus on what happens right after delivery including first few weeks. I believe there is a big focus on pregnancy and stages of labour but very little on what happens next. As a new mum this experience is daunting and if more information is provided beforehand then mum will feel empowered and have resources to reach out if needed…” (multigravida)* *“More talk about after the birth. E.g. how to look after the baby, change nappies, feeding *etc*. Also, how mum may feel and where to find help if needed. When expecting my first baby all the focus was on the birth so I was very prepared but the birth is a very small part of having a baby. What about the days, months and years that follow?” (multigravida)*
Advice and support when birth plans change or unexpected events occur	*“I just want to point out that, despite reservations of midwives regarding talking about the risks of natural birth, a woman has the rights to know about natural birth complications. Consent is not signed for natural birth unlike caesarean but there should be in the very least a more open discussion.*” *(primigravida)* *“I think Input from doctors would be helpful as I have found some other sources to be quite biased towards certain types of deliveries/"natural" births and anti-medical intervention. I feel this can lead to some women feeling like they've failed or are extra frightened if medics end up being involved in delivery” (multigravida)* “*…my first baby died at 37 weeks. At my second baby NCT antenatal classes, stillbirth wasn't mentioned once. The class on when things go wrong was…C-section. When I asked the course why, they said they don't want to scare [people]. That's ridiculous, people need to be informed in a sensible manner.*” *(multigravida)*
Improved mental health support throughout pregnancy	“*There’s not enough information or help for parents to be who struggle with depression and anxiety” (primigravida)* “*Emotional and mental health issues [in] pregnancy can surprise us with relationship issues, money issues, worries” (primigravida)*
The importance of face to face learning	*“There is currently very little face to face support re antenatal care and whatever is provided at present (I.e. phone call appointments) feels rather rushed. I wish that there was a better care given to pregnant women and their partners especially given the current situation with pandemics” (primigravida)* *“I'm currently a bit disappointed that the antenatal classes are online (even though the reasons are very good!) I would like to have someone in person looking with me when I do the practise, to make sure I don't follow the instructions incorrectly without being aware of it…” (primigravida)*
Stage-appropriate delivery of ANE	*“I think the content of antenatal classes needs to be delivered in a manner and format that reduces anxiety- a lot of information I've been given has been in a way which means it is very overwhelming and difficult to take in. The nct class I am due to attend is now suggesting doing the class in one day/ over a weekend and whilst this may be convenient, it does not align with effect[ive] ways of learning and can feel overly intense and overwhelming…” (primigravida)* *“It would be great to be able to choose. E.g., 'I'm at the stage of my pregnancy now where I am thinking about breastfeeding/where to give birth/managing pregnancy discomfort' *etc*. and being able to attend the session that covered that. For different people these issues might be more or less important at different times during their pregnancy.” (primigravida)*

## Discussion

In this study, the majority of participants (79%) do or plan to attend an antenatal class. Women wanted to gain information, skills and make social connections from their antenatal classes. Almost two-thirds of antenatal class attenders wanted to attend more than one class, and this was because of their perception that these different classes meet different needs. When women chose a single class, NHS classes were prioritised. Women who attended NHS antenatal education classes were also more likely to choose exercise classes, suggesting that physical activity is a valued component of their overall preparation for childbirth. This highlights an opportunity for NHS antenatal education to incorporate information on the benefits of exercise during pregnancy and potentially offer guidance or resources for safe physical activity. Overall, women wanted classes to be relevant and appropriate to their stage of pregnancy and to the issues they are currently experiencing or anticipate encountering.

The fact that most women plan to attend NHS classes, underlines the importance of the NHS as a trusted and reliable source of information, and that these classes are a core element of many women’s antenatal preparation. In a recent NHS patient survey conducted by the Care Quality Commission (CQC), fewer than 30% of women said that they were offered NHS antenatal classes or courses, while 29% were not offered any classes and 41% were offered but chose not to attend (Care Quality Commission (CQC), 2020). However, of those who were offered and attended classes, over 90% found them ‘definitely’ useful or useful to ‘some extent’. Although this report did not detail how the classes were offered or what information was provided about their importance, it is vital that antenatal education is accessible to all women and that healthcare trusts find appropriate avenues to ensure awareness and accessibility.

Women attend different types of antenatal class for different reasons. NHS antenatal classes were generally perceived as providing information, whereas exercise classes and private classes were typically sought for social purposes. This is consistent with other studies which confirm the positive impact of social support and connectedness on maternal mental health, reassurance and self-efficacy, as well as the resultant positive impact upon the mother-infant relationship and health of their babies (Feinberg & Kan, 2008; Garthus-Niegel et al., 2018; Leahy-Warren P, 2012; Nolan et al., 2012). Support networks formed in antenatal classes have also been associated with improved attendance and satisfaction with antenatal classes, plus improved postnatal outcomes including reduced rates of depressive symptoms (Ademuyiwa, 2020; Ginja et al., 2018; Smith, 2014). Women largely sought these social connections outside of NHS classes; however, these typically require additional costs, potentially making them less accessible to women from more deprived socioeconomic backgrounds and thus potentially widening existing maternal inequalities and disparities (Ban et al., 2012; Rayment-Jones et al., 2019; Thomson et al., 2013). Inadequate social support is a recognised risk factor for perinatal depression; hence it is essential that antenatal classes facilitate the development of social support networks to enhance maternal self-efficacy and wellbeing in the perinatal period (Elsenbruch et al., 2007; Milgrom et al., 2019).

Women valued opportunities to learn and practice practical skills during antenatal classes. Many of the practical skills women were interested in learning related to labour and birth, for example coping strategies, and postnatal skills including breastfeeding and caring for their newborn. Antenatal classes are an important way for women to develop these skills and prior research has identified the need to counterbalance the information provided to women on labour and delivery, with sufficient focus on the specific realities, challenges and changes brought by the early days of motherhood, particularly for first-time mothers (Barclay et al., 1997). Furthermore, studies have shown that women who did not attend antenatal education were more likely to experience greater worry about labour, and women who regularly attended childbirth preparation classes were less likely to experience fear of childbirth, anxiety or depression (Hassanzadeh et al., 2020; Henriksen et al., 2017; Karabulut et al., 2016; Moghaddam Hosseini et al., 2018). Thus, antenatal education should place greater emphasis on recognising these specific concerns of women and provide information in addition to opportunities to practice these skills, to help women feel more prepared.

Face-to-face classes were valued by participants. This provided opportunities for learning to be practical and guided, to ask professionals or class facilitators questions directly and the environment to meet other women. A recent survey investigating how women preferred to receive information during pregnancy and the postnatal period, revealed that although women commonly used smart phones, or other devices, to source information, most women preferred to receive antenatal education via non-electronic methods (Wright et al., 2020). This remained true across a range of educational, cultural, and socioeconomic levels. The COVID-19 pandemic has resulted in many antenatal classes being made available online, however, our findings coupled with those of previous studies recommends that antenatal classes should return to being face-to-face. It is possible that the context of data collection during the COVID-19 pandemic influenced dissemination and participation in our survey, as well as the strong preference for face-to-face interaction. For the design and development of our antenatal education session (Merriel et al., 2024) the focus groups and co-design occurred prior to the COVID-19 pandemic and thus included opportunities for social interaction. Studies have consistently shown that women highly value social interaction within antenatal education, as it provides a trusted space for sharing experiences and reliable information, and receiving emotional support which can be difficult to replicate in virtual formats (Jones et al., 2019; Spiby et al., 2022). It may be useful to re-examine perspectives on antenatal education provision and expectations, in the post-COVID-19 context. This may be particularly important as, through personal communications with midwifery leaders, we understand that antenatal education has not returned to its pre-COVID-19 state.

Our findings based on antenatal women’s views emphasise the need for a standardised, accessible antenatal education curriculum within the NHS to reduce disparities in access to essential information and skills. Our related survey of antenatal educators revealed similar findings (Russell-Webster et al., 2024). A comprehensive curriculum should include education on the whole spectrum of pregnancy, childbirth, and newborn care, as well as opportunities for practical skill enhancement, including coping strategies during labour and breastfeeding techniques, and focus on supporting perinatal mental health. To meet diverse needs, a flexible delivery model could be developed that can be tailored to individuals and their partners, for example, options for ‘crash courses’, use of digital platforms or hybrid models alongside face-to-face provision, evening sessions or the opportunity to opt into trimester-appropriate sessions. By ensuring NHS antenatal education provision incorporates foundational content, enhances maternal mental health and mitigates social isolation through development of support networks, we can address inequities in educational resources available to expectant parents from a variety of socioeconomic backgrounds.

### Strengths and Limitations

A strength of this study was the online recruitment approach which employed multiple strategies to reach the target population and provided the benefits of quicker enrolment and participation of women participants and greater geographical reach. An additional strength of this study was the use of both qualitative and quantitative methods to aid further, detailed insight into the perspectives of women. However, data acquisition was somewhat limited by the fact that participants frequently did not answer all questions in the survey. The reason for this is not clear.

The women recruited in this study were predominantly White, educated women aged 30 to 39 and residing in England or Wales. Although the survey was distributed nationally with the intention of drawing a representative sample of the UK population, the generalisability of the results may be limited. National UK maternity statistics from Hospital Episode Statistics (HES) showed that in 2021–22 (NHS Digital, 2022), 74% of mothers giving birth in NHS hospitals were White compared to 92.5% in our study. This is important to consider as research has shown that the shortcomings of maternity care provision have greater impacts upon those in marginalised and vulnerable groups within our society, such as those living in extreme financial hardship, from specific ethnic minority groups or those with language barriers (Marmot, 2010; Thomson et al., 2013). For example, Black, Asian and other ethnic minority women are less likely to access high-quality perinatal education and have poorer maternal and child health outcomes (Garcia et al., 2015; Public Health England, 2020). Furthermore, it is noted that cultural insensitivity inadequate consideration of sociocultural norms has been reported as a limitation of antenatal education provision when black African women were asked about their experiences (Esegbona-Adeigbe, 2018). Hence, concerted efforts should be made in further studies to employ methodology that yields a more representative sample of the UK population and captures cultural considerations that improves the accessibility and acceptance of ANE.

Another limitation identified was the unanticipated proportion of women, most of whom were currently pregnant, who did not currently attend or plan to attend an antenatal class. This group was largely comprised of multiparous women, which is consistent with other studies that suggest antenatal education may be less relevant to multiparous women due to factors such as perceived sufficiency of knowledge or potential inaccessibility to due to other commitments (Department of Health, 2011) (Declercq et al., 2007; Stoll & Hall, 2012). Future research to establish what, if any, antenatal education is desired and needed to meet the needs of this group of women would ensure that this large group is not neglected by the NHS provision.

While our study focuses on the experiences of pregnant women, we appreciate the importance of providing antenatal education that is inclusive of and tailored to the partners of pregnant women/birthing people. For example, a recent study highlighted the importance of partner involvement in antenatal education and reported that 82% of birth partners felt more prepared after attending the class (Merriel et al., 2024). Future research should elucidate the perspectives of and antenatal educational needs of partners and antenatal education provision should address barriers to engagement that have been described.

## Conclusions for Practice

Women expect to have access to a comprehensive antenatal programme that equips them with a range of stage-relevant skills and information relevant to pregnancy, labour, and the postnatal period. This can improve birth preparedness and satisfaction, promote the development of perinatal social networks, and ease the transition to parenthood. Free NHS antenatal classes can have a significant positive impact on a woman’s antenatal, birth, and early parenting experience. Our research shows that it is crucial these classes are made more widely available, and that they focus on the needs of the expectant parents. Until this is achieved parents who can afford to will continue to rely on additional private classes, and those for whom these are inaccessible are at risk of poorer experiences and outcomes.

## Supplementary Information

Below is the link to the electronic supplementary material.

Supplementary file1 (DOCX 16 KB)

## Data Availability

Data available on request with appropriate ethical approvals.

## Material Availability

Data available on request with appropriate ethical approvals.
