# Supplementary material for: What Do Antenatal Women Want From Their Antenatal Education? A National Survey
Source: Matern Child Health J. 2025 Feb 3;29(3):324–37. doi: 10.1007/s10995-025-04048-z (PMC11926007; doi:10.1007/s10995-025-04048-z)
Supplement: Supplementary file 1 — Supplementary file1 (DOCX 16 KB) [file 10995_2025_4048_MOESM1_ESM.docx]

**Supplementary data**

***Supplementary Data 1***

**Online questionnaire for women:**

Thank you for taking the time to look at our survey. This is one part of a research study which is trying to design a new antenatal class for women, which will be piloted in Bristol. As part of this we wanted to include the views and experiences of as many women as possible. We would like to understand what information women would like to receive during their pregnancy.

We are therefore inviting women who are currently pregnant, or who are thinking about having a baby to take part in this study. The survey should take no more than 20 minutes of your time.

You will not be identifiable in your answers. You do not need to leave us your contact details at all unless you would like to. However, you will be invited to leave your contact details to be entered into a prize draw for a £50 voucher for Amazon/Love to shop. In addition to this, you will be invited to leave your details if you would like to receive the results of this study.

Your participation is entirely voluntary and you can stop completing the questionnaire at any time and you can withdraw your participation from the study at anytime for 2 weeks after you complete the questionnaire, as long as you have left us your email address.

For further information about the study as a whole please click here (link to what will be the NBT page on the study).

If you have any queries about the study please email xxxxxxxxxxxxx.

If you are willing to participate please click this box to begin:

| 1. Age currently (dropdown all ages 16-55) |
| --- |
| 2. Are you currently pregnant – if yes (to 4, if no to 3) |
| 3. Is your first child? |
| 4. Number of pregnancies:   \| 1 \| 2 \| 3 \| 4 \| 5 \| 6 \| 7 \| 8 \| 9 \| 10 \| Other: how many? \| \| --- \| --- \| --- \| --- \| --- \| --- \| --- \| --- \| --- \| --- \| --- \| |
| 5. Number of children: Dropdown   \| 0 \| 1 \| 2 \| 3 \| 4 \| 5 \| 6 \| 7 \| 8 \| 9 \| 10 \| Other: how many? \| \| --- \| --- \| --- \| --- \| --- \| --- \| --- \| --- \| --- \| --- \| --- \| --- \| |
| 6. Region of residence (drop down menu) |
| 7. Level of education:   \| Pre GCSE \| GCSEs/equivalent \| \| --- \| --- \| \| A-Levels/equivalent \| Bachelors degree/equivalent \| \| Post-graduate degree \| Other \| \| Rather not say \| If other please specify \| |
| 8. Country of origin |
| 9. Ethnicity   \| Asian or Asian British: Bangladeshi \| Black: Any other black background \| White: British \| \| --- \| --- \| --- \| \| Asian or Asian British: Indian \| Chinese \| White: Irish \| \| Asian or Asian British: Pakistani \| Mixed: White and Asian \| White: Any other white background \| \| Asian: Any other Asian background \| Mixed: White and Black African \| Any other ethnic group \| \| Black or Black British: African \| Mixed: White and Black Caribbean \| Not Known \| \| Black or Black British: Caribbean \| Mixed: Any other mixed background \| Prefer not to say \| |
| 10. Are you currently attending or do you plan to attend any antenatal classes? Y / N / DN |
| 11. (If yes to q 10) Do you know what antenatal classes you may attend? (tick all that apply)   \| NHS free classes \| Yoga/pilates \| \| --- \| --- \| \| National Childbirth Trust (NCT) classes \| Aqua aerobics \| \| Hypnobirthing classes \| Other …………….(please state) \| |
| 12. Why do you plan to attend the antenatal classes? (This question will be repeated for each type of antenatal class they state they will attend)   \| To get general information \| To meet people \| \| --- \| --- \| \| To find the answers to specific questions \| To get a general impression \| \| To discuss a specific issue with the midwife \| Other (please state) \| |
| 13. What information are you hoping to gain from the classes   \| Info on labour and delivery \| breastfeeding \| \| --- \| --- \| \| Pain relief \| Understand more about the people involved in providing care during labour and birth \| \| Coping strategies for labour and birth \| Where local services are e.g. \| \| Options for where to have the baby (consultant vs midwife led) \| Practical skills such as Nappy changing, baby bathing \| \| To find out about different options for giving birth to the baby (e.g. vaginal delivery/pool birth/ caesarean/ instrumental delivery) \| To see the labour and birth facilities at the setting \| \| Other (please state) \|  \| |
| 14. Are you trying to learn any practical skills? We have made some possible suggestions below, please tick all that you would like to learn about – and add anything extra in the ‘other’ box, this is not an exhaustive list of what is available but just possible suggestions.   \| Pelvic floor exercise \| Breathing exercises \| \| --- \| --- \| \| breastfeeding \| massage \| \| Hypnobirthing techniques \| Hand expressing \| \| Other… \|  \| |
| 15. During your antenatal classes, who would you like to hear/learn from?   \| Other mothers \| Specialist in the area e.g. hypnobirthing coach \| \| --- \| --- \| \| midwives \| Local charities \| \| doctors \| Other (please state) \| |
| 16. At what stage do you think it would be helpful to start classes?   \| 8-12 weeks \| 13-24 weeks \| 25-35 weeks \| >36 weeks \| \| --- \| --- \| --- \| --- \| |
| 17. do you think you’d like to attend different classes at different points? Yes / no / unsure  If yes – please can you explain what classes you may attend and when? |
| 18. Please rate how important it is for you to receive information about labour and birth at …  This will be displayed as a scale of 1-10   \| midwife appointments \| Extensions of everyday activities e.g. swimming/yoga \| \| --- \| --- \| \| specific antenatal classes \| Home (e.g on phone/computer) \| \| a general mums-to-be informal group \| Other…. \| |
| 19. If you were to be given information by the community midwifery service, in what format do you think it would be best for you to receive it?   \| Information given to me face to face informally \| \| --- \| \| Formal sessions that I attend \| \| Information in printed books/leaflets \| \| Information leaflets on apps/computers \| \| Interactive information e.g. videos \| |
| 20. Do you have any other thoughts about antenatal classes that you would like to share?? |
| 21. Would you like to hear about the results of our study? Yes / No  If yes   \| This survey only \| \| --- \| \| The study as a whole \| \| What is your email address ________________________________ \| |
| 22. Would you like to be entered into our prize draw for a £50 shopping voucher?  If so please leave your email here ______________________________ |
| Thank you for completing the survey  Please could you share the link with friends/family who are about to have or had a baby recently! We’d like to get as many opinions as possible! |
